# Process evaluation of a participatory systems approach to promote health and well-being among students at vocational schools

**DOI:** 10.1093/her/cyaf022

**Published:** 2025-05-31

**Authors:** Clara Heinze, Charlotte Demant Klinker, Anne Sidenius, Rikke Fredenslund Krølner

**Affiliations:** Department of Prevention, Health Promotion and Community Care, Steno Diabetes Center Copenhagen, Borgmester Ib Juuls Vej 83, Herlev 2730, Denmark; National Institute of Public Health, University of Southern Denmark, Studiestraede 6, 1455 Copenhagen, Denmark; Department of Prevention, Health Promotion and Community Care, Steno Diabetes Center Copenhagen, Borgmester Ib Juuls Vej 83, Herlev 2730, Denmark; Department of Prevention, Health Promotion and Community Care, Steno Diabetes Center Copenhagen, Borgmester Ib Juuls Vej 83, Herlev 2730, Denmark; National Institute of Public Health, University of Southern Denmark, Studiestraede 6, 1455 Copenhagen, Denmark

## Abstract

Participatory systems approaches are suggested to address the complex drivers of adolescent health but have not been applied or evaluated in a vocational school setting. This study investigated the implementation of a participatory systems approach to health promotion and its potential for driving system-level changes in vocational schools. We used quantitative data to assess implementation fidelity (reach, recruitment, and dose) and outputs in terms of the potential for system-level changes (engagement, knowledge, and leverage points). Qualitative data examined contextual factors (participant responsiveness, and school and municipal contexts) as potential influences on implementation fidelity and outputs. The results showed that school, municipal, and community actors actively participated in systems mapping and in identifying actions targeting leverage points at various system levels. Engaged and knowledgeable community actors were found to be key in generating ideas targeting the deeper layers of the system, enhancing the potential for successful implementation and system-level change. Implementation fidelity and outputs varied across sites due to varying responsiveness and school and municipal contextual factors. In conclusion, participatory systems approaches in vocational schools are feasible, leading to action ideas with promising leverage points for health promotion. However, a flexible approach tailored to specific school and municipal contexts is needed.

## Introduction

Health and well-being are influenced by many interrelated factors, such as individual aspects (e.g. genetics and behaviour), group dynamics (e.g. connections to friends and family), and physical elements (e.g. urban planning) [1, 2]. Individuals from lower socioeconomic backgrounds are more likely to adopt unhealthy behaviours and experience mental health problems due to, for example, limited access to healthcare, healthy food, and opportunities for physical activity [3, 4]. Across Europe, vocational education schools attract a large number of students from lower socioeconomic backgrounds [5, 6]. Vocational students exhibit significant risk behaviours, such as limited physical activity and poor mental health and well-being, compared with peers in other educational settings [7–10].

Previous health promotion efforts in vocational schools have included programmes targeting physical activity [11], well-being [12], smoking prevalence [13], and sleep habits [14]. Despite the proven effectiveness of such programmes, health promotion efforts in all types of schools are seldom sustained over time [15]. This may be due to limited implementation capacity, staff engagement, financial resources, and the absence of strong networks with other schools, community organizations, or local authorities [15]. The World Health Organization’s Health Promoting Schools (HPS) concept has shown promise in enabling students and staff to take action and sustain health promotion efforts over time [16]. The HPS concept is a whole-school approach designed to address individual, organizational, and policy levels simultaneously, by involving diverse stakeholders (e.g. students, teachers, parents, and the broader community) in developing, implementing, and sustaining change [16]. Whole-school approaches to health promotion can lead to positive outcomes, including improved student health, enhanced academic performance, and a more supportive school culture [17, 18]. However, when developing and implementing such approaches, there is a risk of focusing on inappropriate parts of the school system, leading to unintended negative consequences such as overburdening staff or worsening existing health disparities [19, 20]. Therefore, it is essential to identify modifiable parts of systems (i.e. leverage points) to ensure that actions target the most impactful aspects of the school system, and to develop interventions locally, for example, by addressing students’ needs, staff capacity, and existing school policies and plans [15, 20]. For this purpose, it has been proposed that whole-school approaches to health promotion can be effectively approached through a participatory systems lens, by considering the entire school system and the interconnections among its parts [20–22].

In recent years, system approaches for health promotion have gained significant traction, particularly in community settings [23, 24]. Community-Based System Dynamics (CBSD) represents a structured, promising approach to achieve systems-level changes in communities such as reduction in childhood obesity [25, 26], increase in physical activity [27, 28], and enhancement of healthy food environments [29, 30]. It employs participatory methods to create qualitative causal maps that reveal the underlying structure of a system [31]. Moreover, CBSD seeks to engage diverse stakeholders and codevelop leverage points for actions to change the system in the desired direction [31]. In broader community programmes, diverse schools covering different age groups have often been included as one of several settings to create system changes [e.g. 26,32]. To our knowledge, few studies in primary and secondary educational settings have applied a participatory systems approach, such as CBSD, to advance health-related changes for students [33, 34]. These studies have demonstrated the feasibility of actively involving and engaging both the community and school staff and students, in a participatory systems process to identify key leverage points and develop changes targeting students’ health [33, 34]. However, no studies have focused on vocational schools. Besides the high proportion of students from lower socioeconomic backgrounds in vocational schools, the average age (mean = 24 years) is higher compared with primary and secondary schools. While some students transition directly from primary education at the age of 15–17, almost 40% enrol later in adulthood [8]. These demographic differences, among others, create uncertainty about whether and how a participatory systems approach can be effectively applied in vocational school settings. A process evaluation [35] can help understand if and how a participatory systems approach can be implemented in vocational schools and identify the factors that contribute to success or pose challenges.

The objectives of this study were to (I) assess the implementation fidelity of a participatory systems approach within a vocational school setting; (II) determine the approach’s potential to foster local school and community engagement and knowledge, and identify leverage points with the potential for driving system-level changes (outputs); and (III) examine contextual factors influencing implementation fidelity and outputs.

## Methods

### The vocational school setting

Vocational schools in Denmark combine school education and workplace training to prepare students for specific industries, such as hairdressing, cooking, or electrical work. The programme includes four main tracks: social healthcare, commercial, agriculture and food service, and technical. The number of students per school ranges from 6000 to <100 in multisite versus single-address institutions. Vocational schools are typically organized with one principal manager responsible for overall management, and a teaching and administrative staff. However, multisite schools often have several departmental managers who oversee specific departments [36].

### The participatory systems approach

This study was conducted as part of the evaluation of the Data Health programme (Data-Driven and Systems Approach to Health Promotion among Vocational Students), which has been described in detail elsewhere [37].

Eight vocational school departments and four municipalities (hereafter referred to as eight ‘study sites’) were included in this programme and the current study. The programme applied a participatory cocreation process founded on core principles of data-driven methods [38, 39] and CBSD approaches [31], along with factors that support sustainment in educational settings [15].

Prior to programme activities, each school and municipality appointed a coordinator. Schools received one month’s salary for the coordinator and a handbook outlining responsibilities. Responsibilities included promoting the programme, recruiting committed and motivated participants, and managing practical tasks like data collection. The research team encouraged municipal coordinators to support the schools in recruiting community actors and actively engage in the cocreation process, for example, by offering relevant municipal resources, thereby creating a foundation to support schools in health promotion beyond the programme. In this study, ‘community actors’ are distinct from municipality actors and refer to local or national individuals, organizations, or companies that can provide resources and social support to facilitate the implementation of changes.

To enhance local engagement, the research team asked the schools to identify modifiable health risk factors, such as nicotine use, unhealthy eating habits, physical inactivity, and poor mental health, they wanted to address, as proposed in CBSD approaches [31]. The research team collected and presented survey data on students’ health behaviour and well-being to all students, staff, and managers to guide the identification and selection of the problem. Department managers had the final say but were asked to include insights from data and inputs from both students and staff in their decisions. Four schools decided to focus on students’ engagement in physical activity and four on students’ overall mental health and well-being.

The research team facilitated a group model building (GMB) process consisting of three sessions (GMB1-3) at each school to foster engagement, build capacity, and develop actions targeting leverage points for system-level change. GMB is a qualitative process that involves stakeholders in exploring the origins and contributing factors of an identified problem, visualizing factors and connections on a causal loop diagram (CLD), as well as in identifying leverage points for change [40]. The protocols from the open-access web source ‘Scriptapedia’ [41] were applied to create scripts for GMB1-3 (Table 1). Facilitators were trained in GMB and in using the Systems Thinking in Community Knowledge Exchange (STICKE) software [42] to visualize the CLD by experts at Deakin University [43–45]. School actors (students, staff, and managers) and the municipal coordinator were involved in developing the local CLD during GMB1-2, while the wider community was involved in GMB3. Thus, minor adjustments to the original scripts were made, such as allowing more time for presenting the CLD at GMB3.

**Table 1.** Description of scripts for each GMB session

Session (duration)	Script title	Aim of the script	Participants
GMB1 (2 h)	Introduction and evidence brief	Introduction round; presentation of the GMB process and goals of GMB1; presentation of research related to systems approaches and the chosen complex problem.	Two facilitators School managers, staff, and students Municipal representatives
	Graphs over time	Engage participants in the GMB session by framing the problem, starting the mapping process, and identifying and prioritizing key factors.	
	Connection circles	Map the prioritized factors and the connections and influences between them.	
	Closing	Present the next steps and close the GMB session.	
Between GMB1 and GMB2	Preparation of the CLD	Transcribe the recorded session; the research team adjusts the CLD based on the transcripts from GMB1, categorize the factors into meaningful themes, and highlight leverage points.	The research team
GMB2 (2 h)	Introduction	Present the goals of the sessions.	Two facilitators School managers, staff, and students Municipal representatives
	Model review	Show participants the revised CLD, themes, and leverage points; obtain their feedback on the changes made to the CLD.	
	Model update	Discuss, qualify, and expand the CLD based on personal experiences and understandings, ensuring everyone shares a common understanding of the factors, themes, and leverage points.	
	Identifying relevant stakeholders	Identify and discuss various stakeholders related to the CLD who can assist in the future development and implementation of changes.	
	Closing	Present the next steps and close the GMB session.	
Between GMB2 and GMB3	Preparation of the CLD and prepare the school to recruit participants for GMB3	Transcribe the recorded session; the research team adjusts the CLD based on the transcripts from GMB2, creates a list of possible stakeholders to invite for GMB3, and develops a template for the schools to use for recruitment.	The research team
GMB3 (3.5 h)	Introduction and evidence brief	Introduction round; presentation of the previous process in developing the CLD and goals of the session; presentation of research related to systems approaches and the chosen complex problem.	Two facilitators One notetaker School managers, staff, and students Representation from the municipality Relevant national and community level stakeholders
	Action capture	Identify what is already happening in the school, municipality, or community to address the chosen complex problem.	
	Model presentation and discussion	Present and discuss the CLD; ensure that everyone shares a common understanding of the factors, themes, and leverage points; a notetaker takes notes on minor adjustments to be included in the CLD.	
	Generating action ideas	Generate action ideas for system change on a post it; prioritize actions on a matrix based on their feasibility and potential impact.	
	Sharing action ideas	The top action ideas are shared with everyone; all action ideas are clustered on a large area of wall space.	
	Action groups	Establishment of action groups; each group completes a template with details about their action and creates a preliminary plan for upcoming work and meetings.	
	Closing	Close the GMB session; everyone leaves the session with an awareness of what has been achieved and what will happen next.	
After GMB3	Complete the CLD	The research team adjusts the CLD based on the notes from GMB3, which schools and municipalities can use in future development and implementation of changes.	The research team

*Notes*: GMB: Group Model Building, CLD: Causal Loop Diagram.

### Study design

**Fig. 1.**
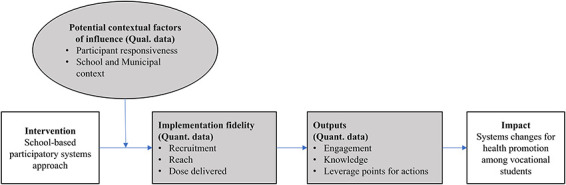
Logic model for the Data Health study with components assessed in the present study indicated by the grey boxes (inspired by the conceptual framework for implementation fidelity [46].

Key process evaluation components (implementation fidelity and outputs of the participatory systems approach, along with contextual factors of influence) were selected by the research group prior to the implementation of the programme. This selection was based on the Medical Research Council’s guidance for process evaluation of complex interventions [35], Carroll and colleagues’ framework for implementation fidelity [46], and key success factors in CBSD approaches [47] (Figure 1), and investigated using qualitative and quantitative methods. Each component was assessed, analysed, and reported independently in the results section, with cross-cutting data patterns explored in the discussion.

Indicators of implementation fidelity included recruitment, reach, and dose delivered, understood as successfully recruiting and reaching the intended target groups with the planned intensity and content. We compared these dimensions to prespecified indicators of preferred practice, based on best practices in CBSD approaches [31], and refined them during the pilot study and protocol development [37]. This resulted in a total of 28 implementation fidelity indicators: six for successful recruitment, 13 for reach, and nine for dose delivered. Desired outputs of the participatory process were operationalized and assessed as levels of engagement and knowledge of all participants, as well as actions targeting leverage points for system-level change.

The context was conceptualized as ‘any feature of the circumstances in which an intervention is conceived, developed, evaluated, and implemented’ [48]. As potential contextual factors influencing implementation fidelity and outputs, we investigated individual- and organizational-level factors [49]. At the individual level, we explored how participants responded to and experienced the process, whereas at the organizational level, we examined school and municipal contextual factors. The data collection methods were selected based on literature recommendations for evaluating processes and implementation fidelity [35, 50–53]. The conceptualization of the components, data collection methods, and preferred practice are presented in Table 2.

**Table 2.** Components, conceptualization, methods, and preferred practice indicators for implementation fidelity, outputs, and contextual factors

Components	Conceptualization	Methods	Preferred practice indicators and categories (bold: preferred practice levels of implementation fidelity for the binary transformation)
**Implementation fidelity**			
Recruitment	*Were the planned procedures for recruiting participants for the GMB sessions followed?*	Semistructured interviews with school coordinators (after GMB2) and municipal coordinators (6 months after GMB3).	*Students*: School coordinator or manager was in charge of recruitment (**Yes**/No). Participated voluntarily (**Yes**/No). *School staff*: School coordinator or manager was in charge of recruitment (**Yes**/No). Participated voluntarily (**Yes**/No). *Community actors*: recruitment was a cooperation between the school and the municipality (**Yes**/No). *P*articipated voluntarily (**Yes**/No).
Reach	*Was the attendance of all participant groups as expected at GMB1-3?*	Notes based on transcripts (GMB1-2) and observations (GMB3)	*GMB1*: 4–10 vocational students (**Yes**/No), 4–10 school staff members (including the school coordinator) (**Yes**/No), 1–2 school managers (**Yes**/No), Minimum 1 from the municipality (including the municipal coordinator) (**Yes**/No). *GMB2*: Same as GMB1. *GMB3*: Same as GMB1 and minimum 5 community actors (**Yes**/No).
Dose delivered	*To what extent was GMB1-3 delivered and implemented consistently and as intended?*	Notes based on transcripts (GMB1-2) and observations (GMB3)	*GMB1*: The session was completed with at least two facilitators (**Yes**/No), The script was followed, and aims met (**Yes**/Minor deviations /Major deviations), The CLD was adjusted after the session (**Yes**/No). *GMB2*: Same as GMB1. *GMB3*: Same as GMB1.
**Outputs**			
Engagement	*Did the participants have the motivation and agency to solve the problem?*	Surveys to all participants after GMB3.	
Knowledge	*Did participants have adequate understanding to solve the problem?*	Surveys to all participants after GMB3	
Action ideas and leverage points	*Could actions be developed targeting various system levels?*	Templates containing detailed descriptions of the action idea (during GMB3).	
**Potential contextual influences**			
Participant responsiveness	*How did participant responsiveness impact implementation fidelity and outputs?*	Semistructured interviews with school coordinators (after GMB2), department managers (after GMB3), and the municipal coordinators (six months after GMB3).	
School and municipality context	*What school- and municipal-level factors had an influence on implementation fidelity and outputs?*	Semistructured interviews with school coordinators (after GMB2), department managers (after GMB3), and the municipal coordinators (six months after GMB3).	

*Notes*: GMB: Group Model Building.

### Data collection and procedures

All data were collected between May 2022 and May 2023. A timeline of data collection activities is provided in Supplementary File 1.

We conducted semistructured interviews with school coordinators (*n* = 8) after GMB2 to explore recruitment practices, responsiveness, and organizational-level influences. We interviewed department managers (*n* = 9, two representing the same school) after GMB3, and municipal coordinators (*n* = 4) 6 months later to further explore contextual influences. All interviews were conducted and recorded online or by phone, lasting about 45 min. To assess the dose delivered and reach, we used transcribed audio files from GMB1 and GMB2 (*n* = 16), and detailed notes from GMB3 (*n* = 8). At GMB1 and GMB2, we determined the number of participants by listening to the introduction round in the audio recordings.

We assessed participant engagement and knowledge through a questionnaire distributed to all GMB3 participants immediately after the session, using a modified version of the Stakeholder-driven Community Diffusion Survey [53]. Engagement was measured with 16 items across five domains: dialogue, flexibility, influence, leadership, and trust. Knowledge was assessed with eight items across five domains: problem, intervention, sustainability, roles, and resources. The responses were scored on five-point Likert scales ranging from strongly agree to strongly disagree. Data on action ideas were collected by having the identified action groups complete a template during GMB3, outlining where and how the desired change should be implemented.

### Data analysis

#### Implementation fidelity

Data on implementation fidelity was analysed by the study site to identify differences. We used content analysis [54] to organize data in relation to the predefined indicators of preferred practice for recruitment, reach, and dose delivered (cf. Table 2). Each component of implementation fidelity was coded as adhering to the preferred practice or not. Overall implementation fidelity scores for each site (0%–100%) were calculated by dividing the number of fulfilled preferred practices by the total of 28 indicators across recruitment, reach, and dose delivered.

#### Outputs

Quantitative data on engagement and knowledge were analysed in SPSS 29.0. Normality was tested using the Shapiro–Wilk test. Descriptive mean scores, between one and five, were reported by the study site and participant group. Engagement was deemed active, and knowledge adequate, with mean scores above 4.0 (agree or strongly agree).

All action ideas were categorized into the four levels of the Action Scales Model (ASM): events, structures, goals, and beliefs [55] to assess their leverage point and, hence, potential for system change when implemented. Our categorization into the four levels was based on definitions provided by the ASM, while also taking related frameworks and understandings into account [56–58]. See Supplementary File 2 for a description of our interpretation of leverage points targeting the four ASM levels. The first author initially categorized action ideas, followed by refinement with all authors until a consensus was reached.

#### Contextual factors

The qualitative interviews were transcribed verbatim and analysed thematically using an inductive approach [59]. Data were analysed separately for each study site and then compared across sites to identify common prominent themes. The first author coded the data, whereafter codes, related relevant dimensions, and corresponding categories were reviewed and refined with two research assistants until consensus was achieved.

### Ethics

The study did not require approval from the Committees on Health Research Ethics for the Capital Region of Denmark (reference number: 22012766), as this is not mandated for social health science research in Denmark [60]. The study was reviewed by the Capital Region of Denmark’s legal centre to ensure compliance with data protection regulations, under journal number P-2021-327. Participation in this study was voluntary, and written informed consent was obtained from all participants prior to conducting interviews, surveys, and observations.

## Results

### Sample characteristics

The participatory systems approach was completed at eight schools across four municipalities during the year 2022: three schools (Schools A–C) between January and June and five schools (Schools D–H) between August and December. Characteristics are summarized in Table 3.

**Table 3.** School characteristics

	School A	School B	School C	School D	School E	School F	School G	School H
Programme start	January 2022	January 2022	January 2022	August 2022	August 2022	August 2022	August 2022	August 2022
Problem selected to be solved	Well-being	Well-being	Physical activity	Physical activity	Physical activity	Well-being	Well-being	Physical activity
Educational track	Social healthcare	Agricultural	Commercial	Technical	Social healthcare	Agricultural	Commercial	Technical
School size	Medium	Small	Small	Large	Large	Medium	Medium	Large
Geographical location (region)	Zealand	Capital region	Zealand	Capital region	Capital region	Zealand	Capital region	Zealand
Municipality	Municipality 1	Municipality 3	Municipality 2	Municipality 1	Municipality 2	Municipality 2	Municipality 3	Municipality 4

*Notes*: School size: small = <800 students, medium = 800–2000 students, Large = >2000 students.

### Implementation fidelity

Table 4 presents results on implementation fidelity and adherence percentages to preferred practice. The overall implementation fidelity varied between 53.3% (School F) and 89.7% (School G).

**Table 4.** Results of the implementation fidelity components (recruitment, reach, and dose delivered)

		School A	School B	School C	School D	School E	School F	School G	School H
**Recruitment**									
Students (GMB1-3)	School coordinator or manager were in charge for recruiting students	**Yes**	**Yes**	**Yes**	**Yes**	**Yes**	**Yes**	**Yes**	**Yes**
	Students participated voluntarily	No	**Yes**	**Yes**	**Yes**	**Yes**	No	**Yes**	No
School staff (GMB1-3)	School coordinator or manager were in charge for recruiting school staff	**Yes**	**Yes**	No	**Yes**	**Yes**	**Yes**	**Yes**	**Yes**
	School staff participated voluntarily	**Yes**	**Yes**	No	**Yes**	**Yes**	**Yes**	**Yes**	No
Community actors (GMB3)	Recruitment of community actors was a cooperation between the school and the municipality	No	**Yes**	No	No	No	No	**Yes**	No
	Community actors participated voluntarily	**Yes**	**Yes**	**Yes**	**Yes**	**Yes**	**Yes**	**Yes**	No
**Reach (number of participants)**									
GMB1	Students	**4**	**4**	**6**	1	**4**	13	13	17
	Staff	3	3	0	2	**6**	2	2	2
	Managers	1	0	**1**	0	0	**1**	**1**	0
	Municipality	**1**	**1**	**1**	**1**	**1**	0	**1**	**1**
GMB2	Students	3	4	5	1	4	15	4	13
	Staff	3	2	0	1	2	2	3	1
	Managers	0	0	**1**	**1**	0	**1**	**1**	0
	Municipality	**1**	**1**	**1**	0	**1**	0	**1**	**1**
GMB3	Students	**8**	**8**	**7**	0	**5**	26	**8**	11
	Staff	1	2	0	3	3	3	1	3
	Managers	**1**	0	0	0	0	0	**1**	**2**
	Municipality	**1**	**1**	**1**	**1**	**1**	0	**2**	**1**
	Community actors	4	**5**	1	4	2	0	**7**	**8**
**Dose delivered**									
GMB1	At least two facilitators	**Yes**	**Yes**	**Yes**	**Yes**	**Yes**	**Yes**	**Yes**	**Yes**
	The script was followed, and aims met	**Yes**	**Yes**	**Yes**	Minor deviations	Minor deviations	Minor deviations	**Yes**	**Yes**
	The CLD was adjusted after the session	**Yes**	**Yes**	**Yes**	**Yes**	**Yes**	**Yes**	**Yes**	**Yes**
GMB2	At least two facilitators	**Yes**	**Yes**	**Yes**	**Yes**	**Yes**	**Yes**	**Yes**	**Yes**
	The script was followed, and aims met	**Yes**	**Yes**	**Yes**	Minor deviations	**Yes**	**Yes**	**Yes**	**Yes**
	The CLD was adjusted after the session	**Yes**	**Yes**	**Yes**	**Yes**	**Yes**	**Yes**	**Yes**	**Yes**
GMB3	At least two facilitators	**Yes**	**Yes**	**Yes**	**Yes**	**Yes**	**Yes**	**Yes**	**Yes**
	The script was followed, and aims met	Minor deviations	**Yes**	**Yes**	Major deviations	**Yes**	Minor deviations	**Yes**	Major deviations
	The CLD was adjusted after the session	**Yes**	**Yes**	**Yes**	**Yes**	**Yes**	**Yes**	**Yes**	**Yes**
Implementation fidelity percent (range 0%–100%)		69.8%	84.5%	70.5%	57.7%	75.3%	53.3%	89.7%	53.6%

*Notes*: Bold indicates the preferred practice was fulfilled. The implementation percent was calculated by dividing the number of fulfilled preferred practices by the total number of 28 indicators.

#### Recruitment

Most schools adhered to preferred practices by having the school coordinator or department manager handle the recruitment of students and staff volunteering to participate. At three schools (Schools A, F, and H), students were obligated to participate, while at one school (School C), the department manager refused to allocate time for staff participation. Only two schools (Schools B and G) fully adhered to the collaborative approach by cooperating with the municipality on recruiting community actors for GMB3, and two schools asked the research team for help in this process (Schools D and E). One school (School H) deviated from the preferred practice by requiring industry representatives from the school’s educational committee to join as community actors for GMB3.

#### Reach

Student participation in GMB sessions was mostly within or above the preferred range of 4–10 participants (mean = 6.7; range 0–26). School staff participation was mainly below the preferred range of 4–10 participants (mean = 2.1; range 0–6), and managers were not present in around half of the sessions. The number of municipal staff generally met the minimum requirement of one per session, whereas nearly two-thirds did not meet the preferred minimum requirement of five community actors participating in GMB3.

#### Dose delivered

At each school, at least two facilitators were present at all three GMB sessions, ensuring consistency in facilitation. At all GMB1 and GMB2 sessions, scripts were followed, and aims were generally fulfilled as planned (cf. Table 1), with minor deviations made at three schools (Schools D, E, and F) such as extending the time for discussions. At two schools (Schools D and H), major deviations occurred for GMB3 as the durations were reduced from 3.5 to 2 h at the schools’ request, resulting in less time for certain activities and aims. For example, at School D, the CLD was completely omitted and only themes from the map were presented. Adjustments to the CLD were made by the research team as planned after each GMB session, using audio files and notes.

### Outputs

Table 5 shows total engagement and knowledge scores for students, staff, managers, municipal representatives, and community actors. For most schools, all participant groups engaged actively (>4.0), except School C (mean = 3.64) and School F (mean = 3.48). Only students scored below this threshold (mean = 3.85). Knowledge scores were below the preferred threshold (>4.0) for all schools, ranging from 3.44 (School F) to 3.91 (School B). Community actors were the only group to exceed the threshold (mean = 4.11), while students scored the lowest (mean = 3.40).

**Table 5.** Results of outputs (engagement, knowledge, and number of actions and their leverage points)

	School A	School B	School C	School D	School E	School F	School G	School H	Total
**Engagement, mean (*N*)**									
Students	3.95 (7)	4.30 (8)	3.51 (7)	N/A	3.93 (5)	3.42 (26)	3.71 (7)	4.36 (11)	3.88
Staff	3.95 (1)	4.93 (2)	N/A	4.32 (3)	4.47 (3)	4.63 (1)	5.00 (1)	4.71 (3)	4.57
Managers	Missing	N/A	N/A	N/A	N/A	N/A	4.21 (1)	4.93 (2)	4.57
Municipality	Missing	4.23 (1)	Missing	4.80 (1)	Missing	N/A	4.40 (2)	3.91 (1)	4.34
Community actors	4.14 (4)	4.48 (4)	4.28 (1)	4.65 (4)	Missing	N/A	4.56 (6)	4.58 (8)	4.45
Total	4.01 (12)	4.43 (15)	3.64 (8)	4.56 (8)	4.13 (8)	3.48 (27)	4.22 (27)	4.50 (25)	4.12
**Knowledge, mean (*N*)**									
Students	3.18 (7)	3.38 (8)	3.09 (7)	N/A	3.71 (5)	3.44 (26)	3.42 (6)	3.60 (11)	3.40
Staff	Missing	4.18 (2)	N/A	3.37 (3)	3.99 (3)	Missing	Missing	4.04 (3)	3.90
Managers	Missing	N/A	N/A	N/A	N/A	N/A	3.50 (1)	3.65 (2)	3.58
Municipality	Missing	3.87 (1)	Missing	3.67 (1)	Missing	N/A	3.43 (2)	3.60 (1)	3.64
Community actors	3.77 (4)	4.22 (4)	4.33 (1)	4.67 (4)	Missing	N/A	4.01 (5)	3.67 (8)	4.11
Total	3.48 (11)	3.91 (15)	3.71 (8)	3.90 (8)	3.85 (8)	3.44 (26)	3.59 (14)	3.71 (25)	3.70
**Number of actions and leverage point**									
Events	2		2		4	2	3	1	14
Structures	2	4				3	4	1	14
Goals			1		1		2	1	5
Beliefs		1	1	1		1	1	1	6
**Total**	4	5	4	1	5	6	10	4	39

*Notes*: N/A: no participants from this group participated.

A CLD formed the basis for identifying leverage points and formulating action ideas that participants perceived as both feasible and impactful across all study sites. An example of a CLD and the related action ideas developed during the programme at one study site (School B) is provided in Supplementary File 3. The number of action ideas varied across the eight study sites, ranging from 1 (School D) to 10 (School G), and with most ideas aligned with the ‘events’ and ‘structures’ levels of the ASM, with fewer targeting ‘goals’ and ‘beliefs’ (Table 5). There was a tendency for students to be involved in action groups targeting leverage points at the event level, while community actors tended to be involved in action groups addressing leverage points targeting the deeper layers of the system (data presented in Supplementary File 4).

### Contextual influences

#### Participant responsiveness

Three themes related to different participant groups’ responsiveness were identified as potentially impacting implementation fidelity and outputs: (I) navigating a complex process, (II) the municipal coordinator role, and (III) student involvement.

Most interviewees expressed challenges in navigating the participatory process but also recognized its value. Managers highlighted that the bottom-up aspect of the process led to long discussions with an unclear focus, which caused frustration. A department manager (School C) remarked, ‘The process [GMB1-3] was important, but a little too long. I think it became a little too democratic perhaps. That annoyed me a bit’, emphasizing the challenge of balancing the open dialogue with a desire to strengthen focus earlier in the process. In addition, varied opinions were expressed about using a CLD to understand the dynamics of a complex problem and generate action ideas. While some found it valuable, such as a school coordinator (School A) who stated, ‘The visual part of seeing how things fit together was really important’, others found the CLD challenging to navigate. For example, a municipal coordinator (Municipality 3) mentioned, ‘I experienced that the systems map flew a bit over the heads of the young people, who were the ones who had to understand it. I actually had a hard time understanding it too.’ Participants’ engagement and ability to contribute effectively may have been affected by these diverse positive and negative perceptions of the process.

Most municipal coordinators mentioned that they would have preferred a more defined role throughout the process. For example, a municipal coordinator from Municipality 4 (coordinator at School H) remarked, ‘I actually think they [the GMB sessions] have been extremely engaging, but I’ve felt a bit like standing on the sidelines. There haven’t really been any requirements for what I was supposed to deliver.’ The lack of defined responsibility may have influenced them negatively as it made municipal coordinators uncertain about their role and the most optimal ways for them to support the process, which potentially has affected their recruitment efforts, diminished their engagement, and their involvement in both the CLD development and in identifying actions.

The involvement of students was widely appreciated, and most interviewees recognized the importance of their perspectives. A school coordinator (School H) indicated, ‘I think it has been really good to involve the young people in the process, as they feel seen and heard in this.’ Similarly, a municipal coordinator from Municipality 3 (coordinator at Schools B and G) remarked, ‘For me it was super interesting and very positive to have the opportunity to sit together with some students and hear their perspectives in this.’ Student involvement might positively have influenced the reach and engagement of staff, municipal, and community actors, as well as the identification of actions that meet the needs of the students.

#### School and municipal context

Three organizational factors emerged that may have impacted implementation fidelity and outputs: (I) management support, (II) resources and competing agendas, and (iii) political prioritization in the municipalities.

Most interviewees emphasized support from the school management as key leaders in fostering engagement in the participatory process. A school coordinator (School E) remarked, ‘Our managers must be role models for us just as much as teachers must be for their students. This [the participatory process] was emphasized and spoken of positively. It was framed as an opportunity, not as an obstacle.’ The same school coordinator (School E) expressed, ‘I don’t think that my manager has given me enough time to do the job [as programme coordinator]. I mean, I don’t want to say, “Right, let’s do this”, knowing that it’s a discount version.’ This discrepancy between the verbal management support for the programme and the practical backing provided in terms of time and resources emerged across several schools.

Limited time and financial resources and the constant competition with educational activities were highlighted as significant barriers to engaging in the GMB process by all interviewees. One school manager (School C) did not allocate time for staff to participate in the process, stating, ‘In terms of resources, you asked us to provide four staff members, etc. I understand that it’s some kind of standardisation, but they [the staff] simply don’t have time in their schedule.’ Student participation was influenced by their frequent alternation between school and workplace-based training, leading some schools to have different students involved in different GMB sessions rather than a consistent group, as proposed. In relation to financial resources, one school manager (School H) highlighted, ‘When a process like this is initiated, it is easy for the students and external actors to create a lot of dreams. However, the students are not always fully realistic about the budgets we are working with; they might have been disappointed.’ Actions were developed and selected based on the participants’ assessment of feasibility and impact. However, the lack of financial resources may have reduced perceived engagement and inclination to select more extensive actions, thereby limiting the potential impact on system-level change.

The political prioritization of health promotion in vocational schools varied across municipalities. The coordinator from Municipality 3 (coordinator at Schools B and G) remarked, ‘At the moment, I actually think that we are better off in terms of resources than they are at the vocational schools. In our municipality, we have the network for health promotion [across all upper secondary educations in the municipality]. So, political prioritisation is there.’ In contrast, coordinators from Municipalities 2 and 4 indicated that they had minimal or no political focus on health promotion within vocational schools. As the coordinator from Municipality 3 was the only one who took an active role during the recruitment of community actors, political prioritization might be an important factor for implementation fidelity.

## Discussion

This process evaluation study explored the implementation fidelity, outputs, and contextual factors of a participatory systems approach aimed at promoting students’ health and well-being in eight vocational schools. Implementation fidelity varied across sites. In terms of outputs, the applied approach successfully engaged actors in developing action ideas and leverage points for system-level change; however, the knowledge levels across most participant groups were relatively low.

Implementation fidelity and outputs appeared to be influenced by various contextual factors, including participant responsiveness to the process and the interaction between the process and the school and municipal contexts. When taking a cross-cutting view of the results, no clear relationship between implementation fidelity and outputs emerged. However, some patterns were identified, and these, along with implications, are discussed below.

### Vocational schools as setting for systems thinking

In alignment with CBSD approaches [31], the Data Health Programme was based on the principle of voluntary participation to ensure the involvement of committed and motivated participants, which is essential for driving systems change. However, in practice, some schools made it compulsory for students to participate. This may be explained by the structured nature of educational institutions in general, which can differ significantly from the more fluid and voluntary nature of community settings [61]. In addition, students had the lowest score of engagement and knowledge compared with other participant groups. In contrast, the qualitative findings indicated that other participant groups valued the involvement of students in the process. Similarly, studies on Youth-led Participatory Action Research [62] demonstrated that other stakeholders highly valued the involvement of schoolchildren and youth in decision-making and it contributed positively to the implementation and sustainability of changes among diverse school settings or communities [63, 64]. Mandating vocational students to participate in the process, despite lower engagement and knowledge levels compared with staff and community actors, might still yield valuable insights for mapping the system and detecting leverage points. However, if students are to be involved in complex systems mapping, the approach may require some adjustments. Previously, youths have been successfully involved in codeveloping system maps through various approaches [65, 66]. McKelvie-Sebileau et al. (2022) separated youth and adults into different sides of the room, allowing them to independently develop their own CLD. Subsequently, the researchers compared and integrated the two maps to gain a comprehensive understanding of the problem from multiple perspectives. This approach encouraged young participants to engage openly and fostered a stronger sense of ownership [65]. Additionally, it might allow facilitators to modify their language, making the tasks more accessible to youths with lower educational backgrounds. In Zambia, researchers adapted GMB sessions for farmers who had limited or no formal education, employing innovative methods to simplify complex concepts. Without access to electricity or computers, the workshop relied on simple hands-on tools such as whiteboards, images of harvested maize, and coins to represent variables [29]. To effectively engage vocational students in a participatory systems process, it is crucial to train facilitators in specific skills, develop targeted techniques, and simplify the complexity of concepts.

### Community and municipal involvement

Literature suggests that involving community actors in diverse school settings can effectively contribute to the implementation and sustainability of health promotion initiatives [5, 34, 67]. Community actors with high levels of engagement and knowledge are better equipped to provide valuable insights, collaborate effectively with other stakeholders, and actively participate in implementing systems change [53, 68]. This study demonstrated that community actors with the necessary knowledge for problem-solving at vocational schools could be successfully engaged. A tendency was observed where community actors were involved in action groups targeting deeper system-level leverage points, such as ‘goals’ and ‘beliefs’, while students were primarily involved in action groups at the ‘events’ level. However, not all sites were able to recruit the desired number of community actors for GMB3. The three sites that successfully recruited more than five community actors either mandated the participation of industry representatives from the schools’ educational committee or collaborated with the municipality to recruit community actors. This suggests that collaboration between vocational schools and municipalities plays a crucial role in involving community actors. In Denmark, most municipalities have clear policies and priorities concerning health promotion in primary schools [67]. However, findings from this study indicated inconsistencies in how municipalities prioritized health promotion at vocational schools. While some municipalities did not prioritize health promotion in vocational schools, others took on the role of intermediary leaders by establishing forums for networking between organizations. An intermediary leader for health promotion can serve many functions, including evaluating impact, aligning and integrating programmes, and promoting supportive policies [67, 69]. We argue, that within health promotion, municipalities might play a valuable role as intermediary leaders, serving as a bridge between the vocational schools and the broader community. A clearer alignment of roles between the school and municipality might have strengthened collaboration, increased community actor involvement, and fostered more impactful leverage points for action. Additionally, given that students spend almost half of their time in workplace-based training, involving representatives from local workplaces in the process could have generated leverage points targeting the broader local system. However, none of the sites chose to recruit representatives from local workplaces. Future research should focus on exploring the potential of involving workplaces as integral participants in system mapping and the identification of leverage points.

### Leverage for system change

A tendency was observed for leverage points to cluster around the ‘structures’ and ‘events’ ASM levels, while fewer ideas addressed the ‘goals’ and ‘beliefs’ levels. This raises concerns about the depth and sustainability of the proposed solutions, as the ‘goals’ and ‘beliefs’ levels present the greatest potential for system change [55]. The four ASM levels were presented briefly at GMB3, where participants were encouraged to codevelop actions based on where they believed they had the most potential to drive change within the system. This suggests the need for future studies to focus on better engaging key leaders in the process with the mandate to work on the deeper layers of the system.

We recommend that future participatory systems processes in schools in general place an even stronger emphasis on applying the ASM or similar frameworks [e.g. 70,71] within the action groups and conduct a systems-level analysis to identify leverage points. This approach was successfully applied within the LIKE programme in a community setting, where action groups utilized the Intervention Level Framework to connect various system levels to detect leverage points over an extended timeframe, facilitated through a series of meetings [72]. Dedicating more time and effort for action groups to identify leverage points, may enable participants to explore the deeper layers of the system. This may facilitate the development of a more cohesive action plan that addresses all levels of the system. Nevertheless, this study demonstrates the feasibility of formulating leverage points targeting the deeper system layers within the planned timeframe and in vocational schools.

### Strengths and limitations

A strength of the study was its practical implications for vocational school policy and the identification of leverage points that could significantly contribute to improving the health and well-being of vocational students. In a subsequent paper, we will explore the implementation of actions and the collaboration process with various system actors during implementation. Anchored in theoretical frameworks, this study offered a methodological approach to conceptualizing implementation fidelity and outputs of a systems approach in school settings. To enhance the credibility and internal validity of the findings, data were collected using multiple methods, as recommended for process evaluations [35]. The generalizability of the process evaluation findings is limited due to contextual differences between the sites involved, such as local municipal prioritization and participants’ prior experiences. However, a notable strength was the design, which involved eight schools across four municipalities, representing various types of vocational schools. This broad geographical and institutional coverage enhanced the relevance of the study and provided a nuanced understanding of how the processes could be implemented in different contexts. To achieve successful implementation across diverse vocational schools, it is essential that the process remains flexible, allowing for adjustments.

A limitation of this study is the lack of perspectives from the students involved. Interviewing students to understand their views on the process could have provided insights into other factors influencing implementation. Furthermore, it could have offered a better understanding of vocational school students’ acceptance of systems approaches for health promotion, which is essential for determining whether vocational students should be involved in future processes. Another limitation is the timing of the interviews with the municipal coordinators, which were conducted six months after GMB3. These interviews also aimed to generate insights into the support for and collaboration with the schools during the implementation of actions. While this timing may have introduced recall bias, it is not seen as a major concern, as the municipal coordinators’ descriptions and perspectives on the process were thorough and detailed.

## Conclusion

This study demonstrated that it was feasible to implement a participatory systems approach in vocational schools and that the process led to the formulation of numerous action ideas with promising leverage points for health promotion. Local school managers, staff, students, municipalities, and community actors actively engaged in systems mapping and identifying leverage points targeting diverse system levels. However, adaptations and further research are needed to better engage students in participatory systems processes within the vocational school setting and to enable participants to generate action ideas addressing the ‘goals’ and ‘beliefs’ levels. Engaged and knowledgeable community actors were found to be key in generating ideas targeting the deeper layers of the system, enhancing the potential for successful implementation and system-level change. Municipalities can play a crucial role in fostering collaborations that connect vocational schools with the broader community to develop system-level changes for health promotion. Implementation fidelity and outputs of the process varied across sites, influenced by diverse contextual factors, underscoring the need for a flexible approach tailored to specific school, municipal, and community settings.

## Supplementary Material

cyaf022_Supp

## Data Availability

The datasets used and analysed during the current study are available from the corresponding author upon reasonable request.

## References

[1] Braveman P, Gottlieb L. The social determinants of health: it’s time to consider the causes of the causes. *Public Health Rep®* 2014;129:19–31. doi: 10.1177/00333549141291S206 PMC3863696 24385661

[2] Lee BY, Bartsch SM, Mui Y et al. A systems approach to obesity. *Nutr Rev* 2017;75:94–106. doi: 10.1093/nutrit/nuw049 28049754 PMC5207008

[3] Zahra A, Lee E-W, Sun L-Y et al. Cardiovascular disease and diabetes mortality, and their relation to socio-economical, environmental, and health behavioural factors in worldwide view. *Public Health* 2015;129:385–95. doi: 10.1016/j.puhe.2015.01.013 25724438

[4] Phelan H, Yates V, Lillie E. Challenges in healthcare delivery in low- and middle-income countries. *Anaesth Intensive Care Med* 2022;23:501–4. doi: 10.1016/j.mpaic.2022.05.004

[5] Pulimeno M, Piscitelli P, Colazzo S et al. School as ideal setting to promote health and wellbeing among young people. *Health Promot Perspect* 2020;10:316–24. doi: 10.34172/hpp.2020.50 33312927 PMC7723000

[6] Milmeister P, Rastoder M, Houssemand C. Mechanisms of Participation in Vocational Education and Training in Europe. *Front Psychol* 2022;13:842307. doi: 10.3389/fpsyg.2022.842307 PMC9190228 35707674

[7] Pisinger V, Thorsted A, Jezek AH et al. *UNG19 - Sundhed Og Trivsel På Gymnasiale Uddannelser 2019*. Copenhagen: Statens Institut for Folkesundhed, SDU, 2019

[8] Ringgaard LW, Heinze C, Andersen NBS et al. *Sundhed Og Trivsel På Erhvervsuddannelser 2019 (UNG19)*. Copenhagen, Denmark: Steno Diabetes Center Copenhagen, Hjerteforeningen og Kræftens Bekæmpelse, 2020.

[9] Atorkey P, Byaruhanga J, Paul C et al. Multiple health risk factors in vocational education students: a systematic review. *Int J Environ Res Public Health* 2021;18:637. doi: 10.3390/ijerph18020637 PMC7828627 33451108

[10] Nelson MC, Larson NI, Barr-Anderson D et al. Disparities in dietary intake, meal patterning, and home food environments among young adult nonstudents and 2- and 4-year college students. *Am J Public Health* 2009;99:1216–9. doi: 10.2105/AJPH.2008.147454 19443824 PMC2696671

[11] Grüne E, Popp J, Carl J et al. Examining the sustainability and effectiveness of co-created physical activity interventions in vocational education and training: a multimethod evaluation. *BMC Public Health* 2022;22:765. doi: 10.1186/s12889-022-13133-9 PMC9011375 35428289

[12] Andersen S, Rod MH, Holmberg T et al. Effectiveness of the settings-based intervention shaping the social on preventing dropout from vocational education: a Danish non-randomized controlled trial. *BMC Psychol* 2018;6:45. doi: 10.1186/s40359-018-0258-8 PMC6134754 30208956

[13] Hjort AV, Christiansen TB, Stage M et al. Programme theory and realist evaluation of the ‘Smoke-Free Vocational Schools’ research and intervention project: a study protocol. *BMJ Open* 2021;11:e042728. doi: 10.1136/bmjopen-2020-042728 PMC7925872 33542044

[14] Vandendriessche A, Deforche B, Dhondt K et al. Combining participatory action research with intervention mapping to develop and plan the implementation and evaluation of a healthy sleep intervention for adolescents. *Health Promot Perspect* 2023;13:316–29. doi: 10.34172/hpp.2023.37 38235009 PMC10790120

[15] Herlitz L, MacIntyre H, Osborn T et al. The sustainability of public health interventions in schools: a systematic review. *Implement Sci* 2020;15:4. doi: 10.1186/s13012-019-0961-8 PMC6945701 31906983

[16] Lee A, Lo A, Li Q et al. Health promoting schools: an update. *Appl Health Econ Health Policy* 2020;18:605–23. doi: 10.1007/s40258-020-00575-8 32291699 PMC7156290

[17] Langford R, Bonell CP, Jones HE et al. The WHO Health Promoting School framework for improving the health and well-being of students and their academic achievement. *Cochrane Database Syst Rev* 2014;4:CD008958. 10.1002/14651858.CD008958.pub2 PMC11214127 24737131

[18] Lee A, Lo ASC, Keung MW et al. Effective health promoting school for better health of children and adolescents: indicators for success. *BMC Public Health* 2019;19:1088. doi: 10.1186/s12889-019-7425-6 PMC6691553 31409312

[19] Ponsford R, Falconer J, Melendez-Torres GJ et al. What factors influence implementation of whole-school interventions aiming to promote student commitment to school to prevent substance use and violence? Systematic review and synthesis of process evaluations. *BMC Public Health* 2022;22:2148. doi: 10.1186/s12889-022-14544-4 PMC9682645 36418997

[20] Rosas SR. Systems thinking and complexity: considerations for health promoting schools. *Health Promot Int* 2015;32:dav109. doi: 10.1093/heapro/dav109 26620709

[21] Daly-Smith A, Quarmby T, Archbold VSJ et al. Using a multi-stakeholder experience-based design process to co-develop the creating active schools framework. *Int J Behav Nutr Phys Act* 2020;17:13. doi: 10.1186/s12966-020-0917-z PMC7006100 32028968

[22] Keshavarz N, Nutbeam D, Rowling L et al. Schools as social complex adaptive systems: a new way to understand the challenges of introducing the health promoting schools concept. *Soc Sci Med* 2010;70:1467–74. doi: 10.1016/j.socscimed.2010.01.034 20207059

[23] Koorts H, Ma J, Swain CTV et al. Systems approaches to scaling up: a systematic review and narrative synthesis of evidence for physical activity and other behavioural non-communicable disease risk factors. *Int J Behav Nutr Phys Act* 2024;21:32. doi: 10.1186/s12966-024-01579-6 PMC10958859 38515118

[24] Nau T, Bauman A, Smith BJ et al. A scoping review of systems approaches for increasing physical activity in populations. *Health Res Policy Syst* 2022;20:104. doi: 10.1186/s12961-022-00906-2 PMC9524093 36175916

[25] Allender S, Millar L, Hovmand P et al. Whole of systems trial of prevention strategies for childhood obesity: WHO STOPS childhood obesity. *Int J Environ Res Public Health* 2016;13:1143. doi: 10.3390/ijerph13111143 PMC5129353 27854354

[26] Coffield E, Nihiser A, Carlson S et al. Shape Up Somerville’s return on investment: multi-group exposure generates net-benefits in a child obesity intervention. *Prev Med Rep* 2019;16:100954. doi: 10.1016/j.pmedr.2019.100954 PMC6706678 31463186

[27] Baugh Littlejohns L, Near E, McKee G et al. A scoping review of complex systems methods used in population physical activity research: do they align with attributes of a whole system approach? *Health Res Policy Syst* 2023;21:18. doi: 10.1186/s12961-023-00961-3 PMC9979563 36864409

[28] Guariguata L, Unwin N, Garcia L et al. Systems science for developing policy to improve physical activity, the Caribbean. *Bull World Health Organ* 2021;99:722–9. doi: 10.2471/BLT.20.285297 34621090 PMC8477427

[29] Kopainsky B, Hager G, Herrera H et al. Transforming food systems at local levels: using participatory system dynamics in an interactive manner to refine small-scale farmers’ mental models. *Ecol Model* 2017;362:101–10. doi: 10.1016/j.ecolmodel.2017.08.010

[30] Mui Y, Ballard E, Lopatin E et al.. A community-based system dynamics approach suggests solutions for improving healthy food access in a low-income urban environment. *PLOS One* 2019;14:e0216985. doi: 10.1371/journal.pone.0216985 PMC6516673 31086409

[31] Hovmand PS. *Community Based System Dynamics*. New York: Springer, 2014. doi: 10.1007/978-1-4614-8763-0

[32] O’Halloran S, Hayward J, Strugnell C et al. Building capacity for the use of systems science to support local government public health planning: a case study of the VicHealth Local Government Partnership in Victoria, Australia. *BMJ Open* 2022;12:e068190. doi: 10.1136/bmjopen-2022-068190 PMC9806011 36572496

[33] Bryant M, Burton W, O’Kane N et al. Understanding school food systems to support the development and implementation of food based policies and interventions. *Int J Behav Nutr Phys Act* 2023;20:29. doi: 10.1186/s12966-023-01432-2 PMC10009978 36907879

[34] Ballard E, Farrell A, Long M. Community‐based system dynamics for mobilizing communities to advance school health. *J Sch Health* 2020;90:964–75. doi: 10.1111/josh.12961 33184879 PMC7702041

[35] Moore GF, Audrey S, Barker M et al. Process evaluation of complex interventions: medical research council guidance. *BMJ* 2015;350:h1258. doi: 10.1136/bmj.h1258 PMC4366184 25791983

[36] Cedefop (ed.), *Vocational Education and Training in Denmark*. Luxembourg: Publications Office of the European Union, 2012

[37] Heinze C, Hartmeyer RD, Sidenius A et al. Developing and evaluating a data-driven and systems approach to health promotion among vocational students: protocol for the Data Health Study. *JMIR Res Protoc* 2024;13:e52571. doi: 10.2196/52571 PMC10879971 38319698

[38] Sigfúsdóttir ID, Thorlindsson T, Kristjánsson AL et al.. Substance use prevention for adolescents: the Icelandic Model. *Health Promot Int* 2009;24:16–25. doi: 10.1093/heapro/dan038 19074445

[39] Crooks N, Strugnell C, Bell C et al. Establishing a sustainable childhood obesity monitoring system in regional Victoria. *Health Promot J Austr* 2017;28:96–102. doi: 10.1071/HE16020 28002719

[40] Siokou C, Morgan R, Shiell A. Group model building: a participatory approach to understanding and acting on systems. *Public Health Res Pract* 2014;25:e2511404. doi: 10.17061/phrp2511404 25828443

[41] Andersen D, Calhoun A, Hovmand P et al. Scriptapedia. 2020. https://en.wikibooks.org/wiki/Scriptapedia. (5 August 2024, date last accessed)

[42] University D. Systems Thinking In Community Knowledge Exchange (STICKE). 2022. https://sticke.deakin.edu.au/.

[43] Maitland N, Wardle K, Whelan J et al. Tracking implementation within a community-led whole of system approach to address childhood overweight and obesity in south west Sydney, Australia. *BMC Public Health* 2021;21:1233. doi: 10.1186/s12889-021-11288-5 PMC8236147 34174853

[44] Allender S, Orellana L, Crooks N et al. Four‐year behavioral, health‐related quality of life, and BMI outcomes from a cluster randomized whole of systems trial of prevention strategies for childhood. *Obesity* 2021;29:1022–35. doi: 10.1002/oby.23130 33950583 PMC8251751

[45] Allender S, Gaskin CJ, Becker D et al. Three-year behavioural, health-related quality of life, and body mass index outcomes from the RESPOND randomized trial. *Public Health* 2024;237:344–53. doi: 10.1016/j.puhe.2024.10.015 39515219

[46] Carroll C, Patterson M, Wood S et al. A conceptual framework for implementation fidelity. *Implement Sci* 2007;2:40. doi: 10.1186/1748-5908-2-40 PMC2213686 18053122

[47] Felmingham T, Backholer K, Hoban E et al. Success of community-based system dynamics in prevention interventions: A systematic review of the literature. *Front Public Health* 2023;11:1103834. doi: 10.3389/fpubh.2023.1103834 PMC10080052 37033017

[48] Skivington K, Matthews L, Simpson SA et al. A new framework for developing and evaluating complex interventions: update of Medical Research Council guidance. *BMJ* 2021;n2061. doi: 10.1136/bmj.n2061 PMC8482308 34593508

[49] Domitrovich CE, Bradshaw CP, Poduska JM et al. Maximizing the implementation quality of evidence-based preventive interventions in schools: a conceptual framework. *Adv Sch Ment Health Promot* 2008;1:6–28. doi: 10.1080/1754730X.2008.9715730 27182282 PMC4865398

[50] Steckler A, Linnan L. *Process Evaluation for Public Health Interventions and Research*. John Wiley & Sons, 2014

[51] Proctor E, Silmere H, Raghavan R et al. Outcomes for Implementation Research: Conceptual Distinctions, Measurement Challenges, and Research Agenda. *Adm Policy Ment Health Ment Health Serv Res* 2011;38:65–76. doi: 10.1007/s10488-010-0319-7 PMC3068522 20957426

[52] Breitenstein SM, Gross D, Garvey CA et al. Implementation fidelity in community‐based interventions. *Res Nurs Health* 2010;33:164–73. doi: 10.1002/nur.20373 20198637 PMC3409469

[53] Korn AR, Appel J, Hammond RA et al. Validation and refinement of the Stakeholder-driven Community Diffusion Survey for childhood obesity prevention. *Implement Sci* 2021;16:91. doi: 10.1186/s13012-021-01158-4 PMC8501696 34627319

[54] Bengtsson M. How to plan and perform a qualitative study using content analysis. *Nurs Plus Open* 2016;2:8–14. doi: 10.1016/j.npls.2016.01.001

[55] Nobles JD, Radley D, Mytton OT. The whole systems obesity programme team. The action scales model: a conceptual tool to identify key points for action within complex adaptive systems. *Perspect Public Health* 2022;142:328–37. doi: 10.1177/17579139211006747 33998333 PMC9720704

[56] Meadows DH. *Thinking in Systems: A Primer*. London: Earthscan, 2009

[57] Harris SG. The fifth discipline: the art and practice of the learning organization, by Peter Senge, New York: Doubleday/Currency 1990. *Hum Resour Manage*. 1990;29:343–8. doi: 10.1002/hrm.3930290308

[58] Malhi L, Ö K, Merth T et al. Places to intervene to make complex food systems more healthy, green, fair, and affordable. *J Hunger Environ Nutr* 2009;4:466–76. doi: 10.1080/19320240903346448 23173029 PMC3489112

[59] Braun V, Clarke V. Using thematic analysis in psychology. *Qual Res Psychol* 2006;3:77–101. doi: 10.1191/1478088706qp063oa

[60] Retsinformation.dk. Lov om videnskabsetisk behandling af sundhedsvidenskabelige forskningsprojekter. 2023. https://www.retsinformation.dk/eli/lta/2011/593#Kap4.

[61] Kwatubana S. School community participation and school health promotion: challenges and opportunities. *Mediterr J Soc Sci* 2014;5:1458–1466. doi: 10.5901/mjss.2014.v5n27p1458

[62] Kohfeldt D, Chhun L, Grace S et al. Youth empowerment in context: exploring tensions in school-based yPAR. *Am J Commun Psychol* 2011;47:28–45. doi: 10.1007/s10464-010-9376-z 21061056

[63] Anselma M, Chinapaw M, Altenburg T. “Not only adults can make good decisions, we as children can do that as well” evaluating the process of the youth-led participatory action research ‘Kids in Action’. *Int J Environ Res Public Health* 2020;17:625. doi: 10.3390/ijerph17020625 PMC7014142 31963706

[64] Kennedy H, DeChants J, Bender K et al. More than data collectors: a systematic review of the environmental outcomes of youth inquiry approaches in the United States. *Am J Commun Psychol* 2019;63:208–26. doi: 10.1002/ajcp.12321 30843254

[65] McKelvie-Sebileau P, Rees D, Tipene-Leach D et al. Community co-design of regional actions for children’s nutritional health combining indigenous knowledge and systems thinking. *Int J Environ Res Public Health* 2022;19:4936. doi: 10.3390/ijerph19094936 PMC9106006 35564331

[66] Savona N, Brown A, Macauley T et al. System mapping with adolescents: using group model building to map the complexity of obesity. *Obes Rev* 2023;24:e13506. doi: 10.1111/obr.13506 36825369

[67] Simovska V, Nordin LL, Madsen KD. Health promotion in Danish schools: local priorities, policies and practices. *Health Promot Int* 2016;31:480–9. doi: 10.1093/heapro/dav009 25753051

[68] Economos CD, Calancie L, Korn AR et al. Community coalition efforts to prevent childhood obesity: two-year results of the Shape Up Under 5 study. *BMC Public Health* 2023;23:529. doi: 10.1186/s12889-023-15288-5 PMC10026415 36941543

[69] Blank MJ. Building sustainable health and education partnerships: stories from local communities. *J Sch Health* 2015;85:810–6. doi: 10.1111/josh.12311 26440823 PMC4606780

[70] Foster-Fishman PG, Nowell B, Yang H. Putting the system back into systems change: a framework for understanding and changing organizational and community systems. *Am J Commun Psychol* 2007;39:197–215. doi: 10.1007/s10464-007-9109-0 17510791

[71] Bolton KA, Whelan J, Fraser P et al. The Public Health 12 framework: interpreting the ‘Meadows 12 places to act in a system’ for use in public health. *Arch Public Health* 2022;80:72. doi: 10.1186/s13690-022-00835-0 PMC8900091 35255970

[72] Luna Pinzon A, Waterlander W, De Pooter N et al. Development of an action programme tackling obesity-related behaviours in adolescents: a participatory system dynamics approach. *Health Res Policy Syst* 2024;22:30. doi: 10.1186/s12961-024-01116-8 PMC10908105 38429775

