# Supplementary material for: Process evaluation of a participatory systems approach to promote health and well-being among students at vocational schools
Source: Health Educ Res. 2025 May 31;40(3):cyaf022. doi: 10.1093/her/cyaf022 (PMC12125708; doi:10.1093/her/cyaf022)
Supplement: cyaf022_Supp [file cyaf022_Supp.zip › Supplementary File 2.doc]

| **DEFINITION (Nobles et al. 2022)** | **Our Understanding of LEVERAGE POINTS** | **Examples from The Data health study** |
| --- | --- | --- |
| **EVENTS:** Aim to suppress the immediate event. They do this by reacting quickly to the visible issues – i.e. ‘quick fixes’. Quite often these actions are needed, but will not address the underlying issues which cause the issue to arise (i.e. the structures, goals and beliefs). | Concrete, visible, and "quick" changes to fix a specific problem. Many "events" are required in order to change the system.  Changes at this level are often easy to identify and establish but may (or may not) require significant resources. | - A cozy student area - A school soccer event - Putting up a wall poster in each class to motivate for PA during breaks - Participate in UngCup (one time event) |
| **STRUCTURES:** Aim to reduce the number or severity of the events occurring. They do this by reshaping or redesigning the organisational or relational system structures, and therefore require an understanding for how the system works. | Attempts to change the mechanisms behind a given problem, including seeking to alter the way things function through adjustments in processes, responsibilities, or collaboration.  Actions at this level may aim to change structures in one part of a system, which in turn affect other parts of the system = feedback loops. | - a peer-to-peer programme - Mental health support (headspace) - Homework assistance at school - Change in submission deadline for assignments. - Mindfulness in class (Teachers should teach positive psychology for a few minutes each day) - Walking bus from the station (weekly) |
| **GOALS:** Aim to re-orientate the goals that the system is working towards. They do this by changing the beliefs of those people setting the system goals. | Goals refer to the specific results a system aims to achieve. There should be a clear focus on clarifying goals by embedding them in for example policies, goal descriptions, or student schedules.  Changing goals adjusts the system's priorities and can redirect effort and resources towards new outcomes. | - Physical activity at the school schedule (physical activity each day) - Establishment of a student council - Physical activity on the agenda for staff meetings |
| **BELIEFS:** Aim to change how individuals and organisations (who influence how the system works) think about the problem. They do this by challenging and changing the deeply held beliefs, norms, attitudes and values within the system. | Seeks to change the shared beliefs, attitudes, and values that create and govern the system. Efforts at this level aim to change attitudes and behavior within the system.  Actions that seek to change the attitudes and values among those who influence the system (mainly staff and managers, as students come and go). | - Training of all teachers as ABC Ambassadors - Leader training ("anføreruddannelses") |

**Supplementary File 2.** Definition by Nobles et al, (2022) and our understanding and examples of leverage points for actions targeting the four levels of the Action Scales Model
