# Supplementary material for: Process evaluation of a participatory systems approach to promote health and well-being among students at vocational schools
Source: Health Educ Res. 2025 May 31;40(3):cyaf022. doi: 10.1093/her/cyaf022 (PMC12125708; doi:10.1093/her/cyaf022)
Supplement: cyaf022_Supp [file cyaf022_Supp.zip › Supplementary File 3.doc]

**Supplementary File 3.** An example of a Causal Loop Diagram from school B, identified leverage points, and action ideas.

**Themes**

*Notes: The solid lines indicate a positive relationship between factors, while the dashed lines indicate a reversed relationship.*
