# Supplementary material for: Process evaluation of a participatory systems approach to promote health and well-being among students at vocational schools
Source: Health Educ Res. 2025 May 31;40(3):cyaf022. doi: 10.1093/her/cyaf022 (PMC12125708; doi:10.1093/her/cyaf022)
Supplement: cyaf022_Supp [file cyaf022_Supp.zip › Supplementary File 4.doc]

**Supplementary File 4.** Description of leverage points for actions, Action Scales Model (ASM) levels and responsible actors.

|  | **Leverage point** | | **Description of leverage points for action** | **ASM level** | **Actors involved in the action group** |
| --- | --- | --- | --- | --- | --- |
| **School A** (targeting students wellbeing as outcome) | Establish an attractive school environment | | Cigarette butts and litter: Setting up trash bins or pots filled with sand to reduce cigarette butts and litter on various paths and parking lots around the school. | Events | Students |
|  | Foster a social culture at the school | | A cosy student area: Create a designated space where students can gather, relax, and socialise across classes and throughout the school, especially during breaks. | Event | Students |
|  | Help new students acclimate to the school's culture | | A peer-to-peer programme: Develop a peer-to-peer programme at the school. An old student should peer up with a new student in the beginning of the semester. | Structures | School staff, municipality |
|  | Strengthen students' beliefs in abilities and capacity to take action | | Mental health support: A formalised collaboration between the school and Headspace. Headspace provides mental health support and resources to help students manage their emotional wellbeing and develop coping strategies. | Structures | Community, school staff |
| **School B** (targeting students wellbeing as outcome) | A more engaging and purposeful commuting to school | | Walking bus: Organise a walking bus from the station (e.g., once a week) to promote physical activity and foster social relationships | Structures | Municipality |
|  | Introduce physical activity during class | | Active breaks: Implement daily active breaks in the classroom by creating a wall poster in each room featuring various examples that teachers should use each day. | Structures | School staff |
|  | A more engaging and purposeful commuting to school | | Carpooling: Putting up a list in the canteen to promote carpooling, as many students drive to school alone. The aim is to save time and money, benefiting the environment, meeting new people. | Structures | Students |
|  | A greater attention to good sleep habits. | | ReloadME materials: Make a plan to utilise ReloadMe materials, in the schools start. The ReloadMe materials is an educational resource for youth focused on sleep habits and digital life. | Structures | Community, school staff |
|  | Increase the experienced support and effectiveness of wellbeing initiatives | | Netwerk programme: Implement the Netwerk programme, which provides teaching materials like lesson plans and films to help students build safe communities, along with activities that foster relationships and seminars to train staff in supporting students facing challenges. | Beliefs | Community, staff |
| **School C** (targeting engagement in physical activity as outcome) | Demand for a social culture at the school | | School soccer event: Organise a school soccer event across different departments at the school. | Events | Students |
|  | Demand for a social culture out of the school | | Activities after school: Involve the student council in organising social activities after school (e.g., bowling). | Events | Students |
|  | Physical activity as an integrated part of the school day | | Physical activity at the school schedule: All first year (GF1) students must be physically active for at least 1 hour each day. This should be included in the school schedule. | Goals | School staff, school manager |
|  | Increase the experienced support and effectiveness of physical activity initiatives | | Build staff capacity: A collaboration with DGI, to build up capacity of staff members to understand the importance of and better manage physical activity during school time. | Beliefs | Community actor |
| **School D** (targeting engagement in physical activity as outcome) | Increase the experienced support and effectiveness of physical activity initiatives | | Leader training ("anføreruddannelsen"): Leader Training "anføreruddannelsen" is a specialised programme in a collaboration with Dansk Firmaidræt. It aims to equip participants with the skills and knowledge needed to initiate and sustain physical activity in schools. | Beliefs | Community actor, School staff |
| **School E** (targeting engagement in physical activity as outcome) | Introduce physical activity during school hours | | Ung-cup: Participate in Ungcup, a nationwide school tournament for vocational educations, organised by Dansk Firma Idræt. | Events | Community actor, students |
|  | Increase student motivation to participate in physical activity | | People's Meeting ("Folkemødet") at Bornholm: A group of students will participate in the People's Meeting at Bornholm (next year) and share their experiences about implementing more physical activity during the school day. | Events | Students, municipality |
|  | Enhance teachers' prioritisation of physical activity during school | | Visibility of existing equipment and activity offers: Create a plan to enhance the visibility of all available equipment and activity offerings for students. | Events | School staff |
|  | Introduce physical activity during class | | Inspiration Catalogue for Movement: A physical copy of an inspiration catalogue providing a variety of exercises and movement ideas that can be easily integrated into the daily routines. | Events | School staff |
|  | Enhance teachers' prioritisation of physical activity during school | | Physical activity on the agenda for staff meetings: Physical activity should be a regular discussion point on the agenda for biannual staff meetings so that teachers can share knowledge: 1) evaluation since the last meeting, 2) new goals and 3) concrete ideas. | Goals | Community actor, Students |
| **School F** (targeting students wellbeing as outcome) | Demand for a social culture at the school | | Excursion with campfire: Organise an Excursion to a Relevant Location such as Jesperhus or Gidselfelt, providing opportunities for students to enjoy campfire activities, including roasting bread, sausages, and marshmallows. | Events | Students |
|  | Demand for a social culture at the school | | A cosy student area: Create a designated space where students can gather, relax, and socialise across classes and throughout the school, especially during breaks. | Events | Students |
|  | Reduce last-minute stress | | Homework Assistance at School: Establishment of homework assistance at school to provide structured support for students needing help with their studies. This programme will feature dedicated spaces utilised during school hours, overseen by staff. | Structures | School staff |
|  | Demand for a social culture at the school | | Peer study groups: Establish peer study groups for new students at the start of each semester. | Structures | Students |
|  | Considering individual needs and life situations | | Mindfulness: Teachers should teach positive psychology for a few minutes each day. Suggested tasks: 1) Students can keep a journal where they note three good things each day. 2) A task to write briefly about themselves and where they see themselves in 10, 20, etc. years. 3) Wake up with a smile. | Structures | Students |
|  | Increase the experienced support and effectiveness of wellbeing initiatives | | ABC for mental health: Training of all teachers as ABC Ambassadors who will promote awareness of the ABC messages related to mental health. The programme will be tailored to the individual school. | Beliefs | Community actor,  school staff |
| **School G** (targeting students wellbeing as outcome) | Demand for a social culture at the school | | School soccer event: Organise a school soccer event across the whole school | Event |  |
|  | Demand for a social culture at the school | | A cosy student area: Create a designated space where students can gather, relax, and socialise across classes and throughout the school, especially during breaks. | Events | Students |
|  | Easy access to cold water | | Water dispensers: Providing students with easy access to drinking water through the installation of water dispensers throughout the school and organise a water-drinking events. | Events | School manager |
|  | Strengthen students' beliefs in abilities and capacity to take action | | Coping skills programme: A coping skills programme, focusing on mental well-being and retention in education, for particularly vulnerable students. In collaboration with the municipal health center. | Structures | School staff |
|  | Help new students acclimate to the school's culture | | Peer study groups: Establish peer study groups for new students at the start of each semester. | Structures | School staff |
|  | Increase the experienced support and effectiveness of wellbeing initiatives | | Me&We: Establishment of a collaboration within the Me&We programme, with the aim to enhance students' social skills and sense of community. The programme will be tailored to the individual school. | Structures | Community actor, school manager |
|  | Easy access to healthy food | | Lower prices in the canteen: Introduce lower prices in the canteen, while ensuring it doesn't compromise the quality of healthy food. | Structures | School manager |
|  | Improve focus during class | | Mobile-free teaching: Explore the possibility of implementing mobile-free teaching, where all students are required to hand in their mobile phones at the start of class and only have access to them during breaks | Goals | School staff, school manager |
|  | Reduce last-minute stress | | Submission deadline for assignments: Changing the deadline for electronic assignments from 11:59 PM to 9:00 PM to encourage timely submissions. | Goals | School manager |
|  | Increase the experienced support and effectiveness of physical activity initiatives | | Leader training ("anføreruddannelsen"): Leader Training "anføreruddannelsen" is a specialised programme in a collaboration with Dansk Firmaidræt. It aims to equip participants with the skills and knowledge needed to initiate and sustain movement and physical activity in schools. | Beliefs | Community actor, school staff |
| **School H** (targeting engagement in physical activity as outcome) | Introduce physical activity during school | | Dedicated room for exercise: Create a dedicated room equipped with table tennis, foosball, a climbing wall, board games, and comfortable sofas to promote physical activity and relaxation among students. | Events | Students |
|  | Introduce physical activity during school | | Physical activity day: Organising a school-wide physical activity day every three months, offering a range of diverse activities. | Structures | School staff |
|  | Participation in decision-making | | Establishment of a student council: Form a student council which should contribute to promoting physical and social activities for students. A staff member should be in charge of organising the counsel and the council will convene monthly or bi-monthly. | Goals | School staff |
|  |  |  | | | |
|  | Increase the experienced support and effectiveness of physical activity initiatives | | Leader training ("anføreruddannelsen"): Leader Training "anføreruddannelsen" is a specialised programme in a collaboration with Dansk Firmaidræt. It aims to equip participants with the skills and knowledge needed to initiate and sustain physical activity in schools. | Beliefs | Community actor |
